# Health care inequality under different medical insurance schemes in a socioeconomically underdeveloped region of China: a propensity score matching analysis

**DOI:** 10.1186/s12889-019-7761-6

**Published:** 2019-10-25

**Authors:** Wei Xian, Xueying Xu, Junling Li, Jinbin Sun, Hezi Fu, Shaoning Wu, Hongbo Liu

**Affiliations:** 1 School of Public Health, China Medical University, Shenyang, People’s Republic of China; 2 Department of Information Center, The Fourth Affiliated Hospital of China Medical University, Shenyang, People’s Republic of China; 3 Simon Fraser University, Burnaby, Canada

**Keywords:** Health care inequality, Healthcare utilization, Healthcare cost, Propensity score, Propensity score matching

## Abstract

**Background:**

Since economic inequality is often accompanied by health inequalities, health care inequalities are increasingly becoming a hot issue on a global scale. As a developing country, China is still facing the same problems as other countries in the world. Especially in underdeveloped regions, owing to the relatively backward economy, health care inequality may be more serious. The objective of this study was to explore health care inequality in a socioeconomically underdeveloped city, thus providing a certain theoretical basis for further development and reform of the medical insurance schemes.

**Methods:**

We mainly extracted relevant insurance information of 628,952 insured enrollees, as well as consumption of outpatient visit and hospitalization. The propensity score matching had been used to estimate different urban medical insurance schemes effect on healthcare utilization, the choice of hospital types and healthcare cost.

**Results:**

Insured enrollees spent most hospitalization expenses in tertiary-level hospitals, which had lowest hospitalization compensation ratios. Healthcare utilization and cost vary significantly by different insurance schemes. Urban employees had significantly higher outpatient visit rates in all hospital types than urban residents. Urban employees preferred to receive hospitalization treatment in tertiary-level hospitals, while those who receive hospitalization treatment in first-level hospitals are more likely to be enrolled in Urban Residents Basic Medical Insurance. Hospitalization expenses and hospitalization compensation ratios of urban employees were also significantly higher than urban residents in all hospital types.

**Conclusions:**

Health care inequality is mainly reflected in the imbalance between hospitalization expenses and hospitalization compensation ratios, as well as inequalities under different medical insurance schemes in healthcare utilization, the choice of hospital types and healthcare cost in socioeconomically underdeveloped regions of China. We should conduct a targeted medical insurance reform for the socioeconomically underdeveloped regions, rather than applying templates of ordinary regions. Further efforts are needed in the future to provide equal health care for every patient.

## Background

Since economic inequality is often accompanied by health inequalities, health care inequalities are increasingly becoming a hot issue on a global scale [1–4]. As people pay attention to their own health, health care inequalities are getting more and more attention from the public. As a developing country with fast economic growth, China also faces the same problems as other countries in the world. According to the World Health Report in 2000, China’s overall health system performance ranked 144 out of 199 countries and the ranking of equity in health financing was third from last in the world [5]. There are two main reasons for this phenomenon. One of them is insufficient coverage of medical insurance [6], and the other is health care inequality. In the past 20 years, China has achieved a series of health reforms [7, 8]. It included further coverage of Urban Employees Basic Medical Insurance (UEBMI) and Urban Residents Basic Medical Insurance (URBMI). UEBMI is a social insurance system established in 1998 to compensate urban employees for economic losses due to disease risks. However, the elderly, children, students, and urban non-employed residents are not included in this scheme. Therefore, in order to reduce the medical expenses brought by the growing health needs of residents, URBMI has been implemented since 2007. The implementation of URBMI indeed makes up for the health care of some groups and improves health care inequalities. The coverage rate of medical insurance in China has exceeded 95% [9, 10]. But does this fast-developing insurance system mask undetectable health care inequalities and fail to achieve real health care inequalities? With the coverage of universal medical insurance in China, health care inequality has become more important and needs to be taken seriously. Health care inequality is mainly reflected in the imbalance between hospitalization expenses and hospitalization reimbursement, as well as inequalities under different medical insurance schemes in the use of medical resources and the choice of hospital types.

In today’s China, citizens can directly go to any level of hospital, but they are still more willing to choose high-level hospitals for treatment [11]. In order to alleviate the pressure on high-level hospitals, China has implemented grading diagnosis and treatment system, including a two-way referral system, to leave some patients with slight illness in low-level hospitals. However, it still cannot completely eliminate the phenomenon of “inverted pyramids” in China. In some cities, there are still many patients gathered in high-level hospitals to see a doctor [12]. Patients believe that they can get relatively better treatment in high-level hospitals [11]. In addition to focusing on better treatment, patients are also very concerned about medical expenses. So does the phenomenon of “inverted pyramids” exist in socioeconomically underdeveloped regions? Does the hospital with the most hospitalization expenses have the most hospitalization reimbursement? These issues are worthy exploring further.

Although the use of health care should be distributed equitably based on people’s needs rather than ability to pay [13], the current use of health care is unequal. Healthcare utilization is more beneficial to the rich [14]. The rich enjoy the relatively high-level healthcare utilization, while the poor have to choose lower-level services or even no money to get health care [15, 16]. So there is such a saying that it is difficult and expensive to see a doctor. Underdeveloped regions are relatively backward in economy. In these regions, the difficulty and high expense for getting medical treatment may be more serious. Therefore, the use of medical resources and the choice of hospital types may be different from those in ordinary regions and require more attention. Since different insurance schemes have an impact on healthcare utilization and costs [17, 18], it is worth considering whether different insurance schemes will have a greater impact on healthcare utilization, the choice of hospitals and healthcare cost in socioeconomically underdeveloped regions. Earlier studies focused more on ordinary regions and did not use propensity scores to reduce the effects of confounding factors [19–22], which had increased bias in the research.

Medical inequality is easily hidden by economic development, so our aim was to explore whether there is an imbalance between hospitalization expenses and hospitalization reimbursement, and whether there are inequalities under different medical insurance schemes in healthcare utilization, the choice of hospital types and healthcare cost in a socioeconomically underdeveloped city of China. Our paper contributes to a certain theoretical basis for policy makers to pay more attention to health inequalities, thus eliminating the greater hidden dangers in the future.

## Methods

### Study setting

Fuxin city is the typical socioeconomically underdeveloped city in China. The economy of Fuxin city is relatively backward compared to ordinary cities in China [23]. The urban population is 1.04 million. About 70% of them participate in UEBMI or URBMI.

### Data source

The data in this study were derived from Fuxin Social Insurance Bureau. Fuxin Social Insurance Bureau collected all medical insurance data annually from all insured enrollees in Fuxin city. Data related to insured information will be updated at any time. We mainly extracted relevant insurance information of insured enrollees in 2015, as well as consumption of outpatient visit and hospitalization.

UEBMI enrollees were aged 17 and older, while URBMI enrollees included many infants and children. However, infants and children were susceptible to various diseases, often accompanied by greater medical expenses compared to people of other ages. We excluded this small proportion of insured enrollees to prevent differences in medical expenses and age differences after matching. Therefore, insured enrollees aged 17 and older became the research subjects of this study.

The demographic characteristic and medical expenses of insured enrollees were extracted. The demographic characteristic mainly included gender, date of birth, workplaces (enterprises, individual, state-owned workplaces and other workplaces), and wages (low wages, medium wages, high wages). We defined ages into three groups: 17–45, 45–60, and 60-. According to average social wages of employees in Fuxin city, the wages are divided into three levels: low, medium and high. In addition, we collected the dates of visit, hospital types, out-of-pocket expenses, insurance expenses, and total expenses related to outpatient visit and hospitalization.

### Statistical methods

The propensity score was first proposed by Rosenbaum and Rubin in the 1980s [24]. Propensity score matching (PSM) is to make the selected two groups comparable in terms of potential confounding factors, in order to balance variables and reduce bias. In the first step of PSM analysis, a series of demographic variables which might impact on healthcare utilization, hospital types and healthcare cost were considered to calculate the propensity score. The selected variables were based on data availability and literature review, including gender, age, workplaces, and wages. We performed multivariate logistic regression to calculate the propensity score. In the second step of PSM analysis, Greedy matching techniques, proposed by Parsons [25], was chosen to perform one-to-one matching. After matching, the quality of the match was verified by calculating standardized bias and conducting a significance test. Standardized bias are calculated by the formula,

$$ d=\frac{100\ast \left({p}_{1-}{p}_2\right)}{\sqrt{\frac{p_1\left(1-{p}_1\right)+{p}_2\left(1-{p}_2\right)}{2},}} $$

where *p*_1_ represents the group of URBMI enrollees and *p*_2_ represents the group of UEBMI enrollees. When standardized bias is less than 0.1, the covariates between the two groups are balanced and there is no difference between them.

The chi-square test was adopted to estimate different medical insurance schemes effect on healthcare utilization and the choice of hospital types. Hospital types are divided into three levels, namely the first-level hospital, the secondary-level hospital and the tertiary-level hospital. First-level hospitals correspond to low-level hospitals, and tertiary hospitals correspond to high-level hospitals. Non-parametric test was used to assess the impact of different medical insurance schemes on healthcare cost. We mainly used hospitalization expenses and hospitalization compensation ratio as a measure of healthcare costs. Hospitalization compensation ratio refers to the ratio of the amount reimbursed by medical insurance to the total hospitalization expenses.

All indicators were calculated using SAS software, version 9.4. None of the funders had any role in the design of the study and collection, analysis, and interpretation of data and in writing the manuscript.

## Results

### Demographic characteristics of all insurance enrollees

There were 628,952 people participating in medical insurance, including 549,603 UEBMI enrollees and 79,349 URBMI enrollees. Table 1 reported the summary statistics of key variables. The results indicated the trends of hospitalization expenses and hospitalization compensation ratios in different hospital types were completely opposite. Insured enrollees spent most hospitalization expenses in tertiary-level hospitals, which had lowest hospitalization compensation ratios. However, first-level hospitals with highest hospitalization compensation ratios had the lowest hospitalization expenses. This phenomenon had appeared in both insurance schemes. It is not difficult for us to find an unreasonable phenomenon. That is, there has been an imbalance between hospitalization expenses and hospitalization compensation ratios in a socioeconomically underdeveloped region.

**Table 1:** Summary statistics of key variables (*N* = 628,952)

		UEBMI enrollees (*N* = 549,603)	URBMI enrollees (*N* = 79,349)
Basic demographic variables (number/percent)			
Age (years)	17–45	180,430 (32.83%)	23,832 (30.03%)
	45–59	229,889 (41.83%)	26,501 (33.40%)
	> 59	139,284 (25.34%)	29,016 (36.57%)
Gender	Male	276,043 (50.23%)	30,576 (38.53%)
	Female	273,560 (49.77%)	48,773 (61.47%)
Workplaces	Enterprise	273,185 (49.71%)	345 (0.43%)
	Individual	180,548 (32.85%)	282 (0.36%)
	State-owned workplaces	92,593 (16.85%)	78,717 (99.20%)
	Other workplaces	3277 (0.60%)	5 (0.01%)
Wages	Low	354,049 (64.42%)	63,658 (80.23%)
	Medium	42,355 (7.71%)	2958 (3.73%)
	High	153,199 (27.87%)	12,733 (16.05%)
Outpatient visit (%)			
Any outpatient visit in the past year		17.47	1.14
Hospital types	First-level hospital	9.66	0.10
	Secondary-level hospital	2.12	0.94
	Tertiary-level hospital	8.41	0.14
Hospitalization (%)			
Any hospitalization in the past year		14.09	12.20
Hospital types	First-level hospital	1.73	1.31
	Secondary-level hospital	4.92	4.87
	Tertiary-level hospital	8.72	7.12
Hospitalization expenses (median/ lower quartile ~upper quartile, RMB)			
Hospital types	First-level hospital	3075.10 (2183.99~4934.57)	2990.65 (1919.77~4610.90)
	Secondary-level hospital	4611.98 (2942.49~7294.23)	4002.23 (2467.00~6689.57)
	Tertiary-level hospital	6085.53 (3979.50~10,396.24)	5503.85 (3491.24~9630.11)
Hospitalization compensation ratios (median/lower quartile ~upper quartile, %)			
Hospital types	First-level hospital	77.81 (73.76~82.35)	69.81 (63.20~81.81)
	Secondary-level hospital	76.36 (71.50~81.31)	70.02 (62.03~81.25)
	Tertiary-level hospital	67.81 (62.86~72.54)	58.74 (52.24~72.15)

### Match evaluation

In order to better compare different insurance schemes, it is necessary to balance basic demographic variables. Table 2 showed the multivariate logistic regression results for propensity scores. Age, gender, workplaces, and wages between UEBMI enrollees and URBMI enrollees were statistically significant. A one-to-one match was made based on the generated propensity scores. The final sample size for analysis was 36,031 UEBMI enrollees and 36,031 URBMI enrollees.

**Table 2:** Multivariate logistic regression results for propensity scores

	Estimate	Standard Error	Odds Ratio (95% Confidence Interval)
Age (years)			
17–45	Reference		
45–59	−0.404	0.015	0.668 (0.648, 0.688)**
> 59	1.103	0.017	3.014 (2.913, 3.118)**
Gender			
Male	Reference		
Female	−0.608	0.012	0.545 (0.532, 0.558)**
Workplaces			
Enterprise	1.066	0.452	2.905 (1.198, 7.043)*
Individual	1.887	0.453	6.597 (2.717, 16.022)**
State-owned workplaces	−6.350	0.449	0.002(< 0.001, 0.004)**
Other workplaces	Reference		
Wages			
Low	Reference		
Medium	2.352	0.026	10.504 (9.991, 11.043)**
High	3.256	0.016	25.946 (25.126, 26.794)**

Note: *p < 0.05, **p < 0.01

The PSM improved comparability between UEBMI enrollees and URBMI enrollees. Standardized bias for all variables between UEBMI enrollees and URBMI enrollees were lower than 5% after matching. By matching, all variables were balanced between the two groups. In addition, on the basis of the chi-square test, the difference between UEBMI enrollees and URBMI enrollees was statistically insignificant for all characteristics (*p* > 0.05). Detailed PSM evaluation results were shown in Tables 3.

**Table 3:** Comparison of sample characteristics before and after propensity score matching (PSM)

	Before PSM				After PSM			
	UEBMI (%)	URBMI (%)	Standardized Bias (%)	*P* value	UEBMI (%)	URBMI (%)	Standardized Bias (%)	*P* value
Age (years)								
45–59	41.83	33.40	−17.47	< 0.001	34.12	34.02	−0.21	0.777
> 59	25.34	36.57	24.47	< 0.001	39.81	39.89	0.16	0.831
Female	49.77	61.47	23.71	< 0.001	60.95	60.92	−0.06	0.927
Workplaces								
Enterprise	49.71	0.43	− 138.21	< 0.001	0.95	0.96	0.10	0.970
Individual	32.85	0.36	−97.04	< 0.001	0.79	0.78	−0.11	0.966
State-owned workplaces	16.85	99.02	302.68	< 0.001	98.25	98.25	0	1.000
Wages								
Medium	7.71	3.73	−17.20	< 0.001	8	8.21	0.77	0.306
High	27.87	16.05	−28.85	< 0.001	35.55	35.34	−0.44	0.559

### The effect of medical insurance schemes on healthcare utilization

Table 4 presented outpatient visit rates and hospitalization rates for different hospital types under different medical insurance schemes. We can see that any outpatient visit in the past year of UEBMI enrollees and URBMI enrollees were statistically different. Among all hospital types, UEBMI enrollees had significantly higher outpatient visit rates than URBMI enrollees. Insured enrollees in the UEBMI scheme increased the use of outpatient services. There was also a statistical difference in any hospitalization in the past year between the two groups. However, hospitalizations of both groups were only statistically different in first-level and tertiary-level hospitals. UEBMI enrollees had significantly higher hospitalization rate in tertiary-level hospitals, while hospitalization rate of URBMI enrollees was higher in first-level hospitals. Compared with the possibility of URBMI enrollees being hospitalized, UEBMI enrollees were more likely to receive treatment in tertiary-level hospitals rather than in first-level hospitals.

**Table 4:** Effect of different medical insurance schemes on healthcare utilization

		UEBMI (%)	URBMI (%)	χ^2^ statistics	*P* value
Outpatient visit					
Any outpatient visit in the past year		31.15	1.17	11,950.495	< 0.001
Hospital types	First-level hospital	13.62	0.12	5125.879	< 0.001
	Secondary-level hospital	4.25	0.93	785.221	< 0.001
	Tertiary-level hospital	18.85	0.15	7327.862	< 0.001
Hospitalization					
Any hospitalization in the past year		14.95	13.36	37.105	< 0.001
Hospital types	First-level hospital	1.26	1.51	8.422	0.004
	Secondary-level hospital	4.52	4.73	1.865	0.172
	Tertiary-level hospital	10.09	8.37	63.467	< 0.001

### The effect of medical insurance schemes on healthcare cost

**Fig. 1:**
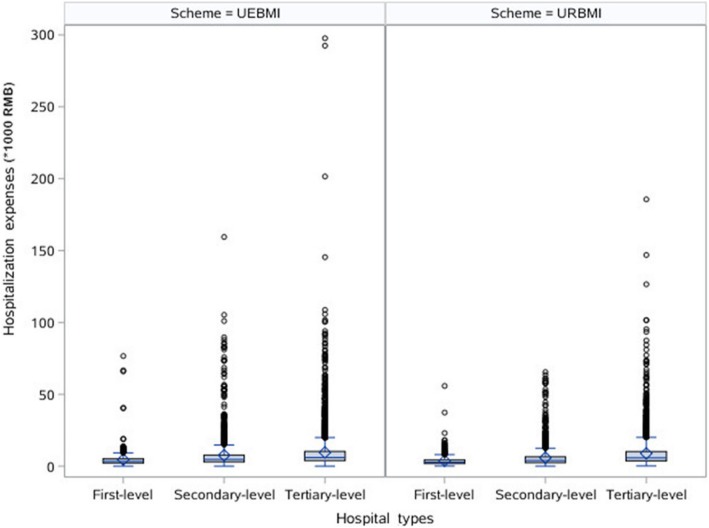
Hospitalization expenses for different hospital types under different insurance schemes (Comparison of UEBMI enrollees and URBMI enrollees: first-level hospital, *Z* = 3.931, *p* < 0.001; secondary-level hospital, *Z* = 6.824, *p* < 0.001; tertiary-level hospital, *Z* = −2.133, *p* = 0.033)

**Fig. 2:**
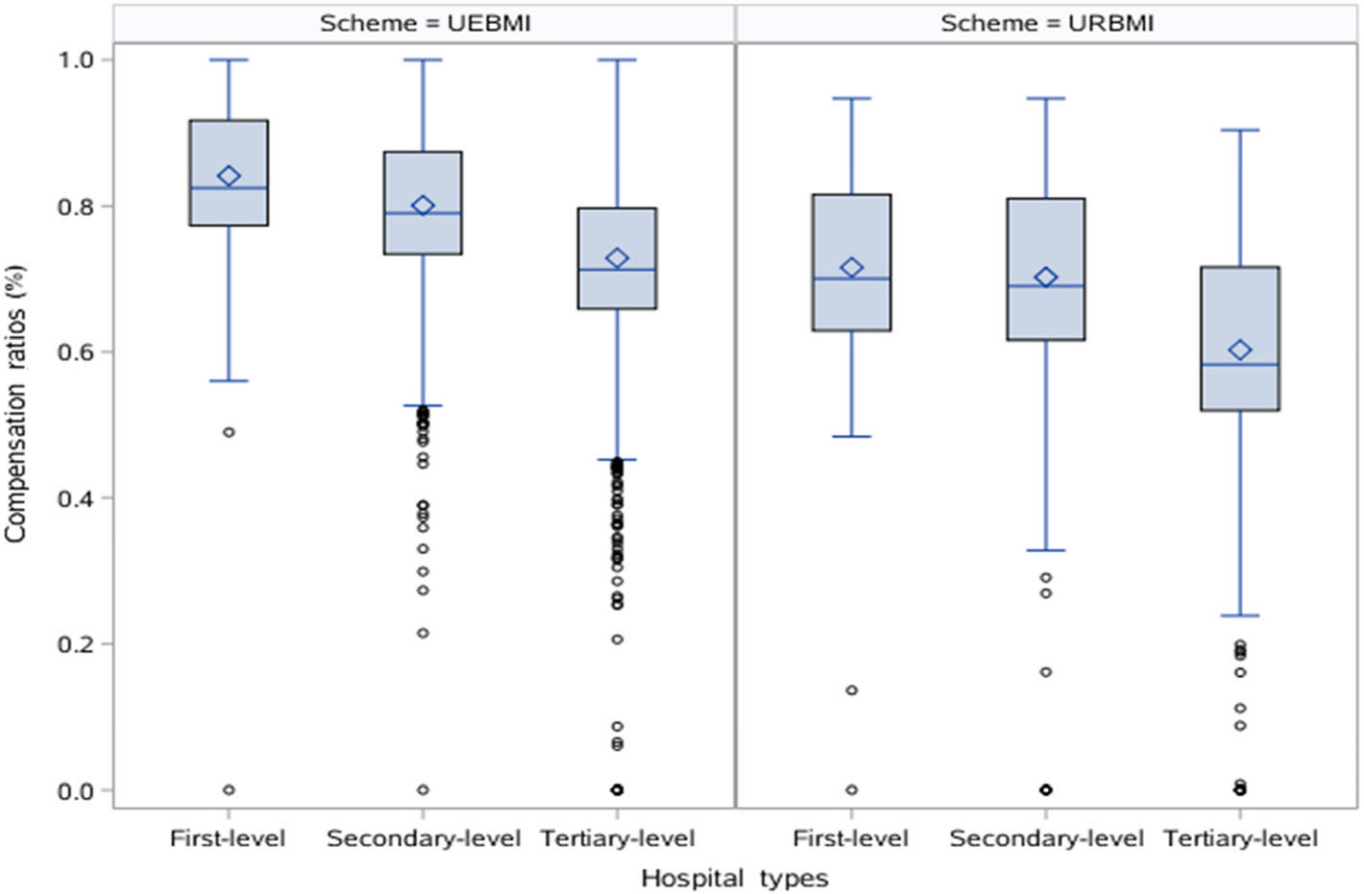
Hospitalization compensation ratios for different hospital types under different insurance schemes (Comparison of UEBMI enrollees and URBMI enrollees: first-level hospital, *Z* = 15.366, *p* < 0.001; secondary-level hospital, *Z* = 21.266, *p* < 0.001; tertiary-level hospital, *Z* = -36.077, *p* < 0.001)

Figures 1 and 2 showed the distribution of hospitalization expenses and hospitalization compensation ratios for different hospital types under different insurance schemes. No matter what hospital types, UEBMI enrollees and URBMI enrollees had statistically significant differences in hospitalization expenses and hospitalization compensation ratios. Furthermore, hospitalization expenses and hospitalization compensation ratios of UEBMI enrollees were both higher than those of URBMI enrollees in all hospital types.

## Discussion

Providing equal health care to every patient is the goal of all countries around the world. China is also making unremitting efforts to this end. In the past, China has made some progress in providing affordable and equitable basic medical services to the population, which has surpassed many developing countries. However, health care inequalities enjoyed by each subgroup of insurance enrollees still exist. This paper indicated the imbalance between hospitalization expenses and hospitalization reimbursement, and systematically estimated the impact of different medical insurance schemes on health care inequality in healthcare utilization, the choice of hospital types and healthcare cost in a socioeconomically underdeveloped city.

In a socioeconomically underdeveloped city, the phenomenon of “inverted pyramids” still exists. In the inherent misconceptions of many Chinese people, the need for hospitalization indicates a very serious disease, and high-level hospitals can get good treatment. At this time, people wanted to get the best treatment. Our results showed that first-level hospitals had highest hospitalization compensation ratios. But it was not the first thing they considered. As a result, most hospitalization expenses are still spent in tertiary-level hospitals. This is an abnormal phenomenon that leads to a failure to better allocate and utilize medical resources, thus causing a waste of medical resources. In terms of the choice of hospital types, UEBMI enrollees had higher outpatient visit rates in all hospital types than URBMI enrollees (*p* < 0.001). URBMI enrollees were less likely to use outpatient visit services, probably because URBMI enrollees were unable to get compensation from the insurance scheme and can only use their own money for outpatient visits. The money they have can only guarantee their basic life. So they usually try to minimize the use of outpatient visit services. Our results also indicated that UEBMI enrollees were more willing to receive hospitalization treatment in tertiary-level hospitals (p < 0.001), while those who receive hospitalization treatment in first-level hospitals are more likely to be enrolled in URBMI (*p* = 0.004). We can see that insured enrollees under different insurance schemes have inequalities in the choice of hospital types due to economic inequalities. However, this result is inconsistent with Zhou, et al. [26]. Zhou et al. [26] thought that UEBMI enrollees in Shaanxi Province may be more willing to go to lower-level hospitals for hospitalization. This further explains that the imbalance of hospitalization expenses and hospitalization compensation ratios may be more obvious in a socioeconomically underdeveloped city.

Healthcare utilization varies significantly by different insurance schemes. Insured enrollees in the UEBMI scheme had higher likelihood of healthcare utilization. This may be due to URBMI enrollees have relatively low incomes compared to the UEBMI enrollees. Only when URBMI enrollees have relatively serious illnesses can they go to the hospital for treatment. It showed that the inequalities in the use of medical resources are largely not based on differences in demand but on the economy. Although different insurance schemes target different groups, if there are always huge differences between different insurance schemes, this will bring greater hidden dangers in the future.

Inequalities in healthcare utilization also have left a huge mark on healthcare cost [27]. UEBMI enrollees had higher hospitalization expenses in any hospital type (*p* < 0.05). In addition, hospitalization compensation ratio of UEBMI enrollees was also significantly higher than that of URBMI enrollees (*p* < 0.001). Our findings are consistent with Zhang, et al. [28] in that UEBMI enrollees have the highest compensation ratios and compensation ratios of medical insurance vary significantly by schemes. From this perspective, there is no difference in the hospitalization compensation ratio between socioeconomically underdeveloped regions and ordinary regions. This may be due to the different reimbursement of different urban medical insurance schemes, which also reflects the existence of health care inequality and needs to be valued by government.

Similar to Dou et al. and Yu et al. [29, 30], we conclude that although China has successfully achieved the goal of providing medical insurance for almost the entire population, it faces the challenge of health care inequality. The challenge still continues, and policymakers should now make efforts to reduce health care inequality between subgroups of insurance enrollees. We offer some advices on the current medical insurance policy in socioeconomically underdeveloped regions. The grading diagnosis and treatment system should be further implemented to balance hospitalization expenses and hospitalization compensation ratios. Slight illnesses go to low-level hospitals, and then go to higher-level hospitals. If insurance enrollees participate in this system, the medical insurance reimbursement will increase relatively, and if not, it will fall. We also need to strengthen publicity, improve people’s awareness, and make health resources more rationally allocated and utilized. In order to achieve an equality of insurance benefits under different insurance schemes, a large amount of additional public funds is needed. The government can subsidize socioeconomically underdeveloped regions to expand the redistribution of resources.

There are some limitations in our study. By using matching techniques to increase the comparability between different insurance schemes, unobservable (unmatched) characteristics may affect the presented results. During the investigation, we collected data as comprehensively as possible. Completely ideal data is difficult to obtain. In addition, our study did not collect information on the existence of chronic diseases, the presence of special occupational diseases, pregnancy status, etc., which may affect the demand for healthcare utilization.

## Conclusions

Healthcare utilization varies significantly by different insurance schemes. Insured enrollees in the UEBMI scheme had higher likelihood of healthcare utilization. Hospitalization expenses and hospitalization compensation ratios of UEBMI enrollees were also significantly higher than those of URBMI enrollees. Health care inequality is mainly reflected in the imbalance between hospitalization expenses and hospitalization compensation ratios, as well as inequalities under different medical insurance schemes in healthcare utilization, the choice of hospital types and healthcare cost in socioeconomically underdeveloped regions of China. We should conduct a targeted medical insurance reform for the socioeconomically underdeveloped regions, rather than applying templates of ordinary regions. Further efforts are needed in the future to provide equal health care for every patient.

## Data Availability

The datasets used and analyzed during the current study are available from the corresponding author on reasonable request.

## Abbreviations

PSM: Propensity score matching
UEBMI: Urban Employees Basic Medical Insurance
URBMI: Urban Residents Basic Medical Insurance
